# The integrated stress response in metabolic adaptation

**DOI:** 10.1016/j.jbc.2024.107151

**Published:** 2024-03-09

**Authors:** Hyung Don Ryoo

**Affiliations:** Department of Cell Biology, New York University Grossman School of Medicine, New York, New York, USA

**Keywords:** integrated stress response, amino acid deprivation, mitochondrial stress, GCN2, GCN1, HRI, eIF2α, ATF4, serine biosynthesis, cysteine, glutathione

## Abstract

The integrated stress response (ISR) refers to signaling pathways initiated by stress-activated eIF2α kinases. Distinct eIF2α kinases respond to different stress signals, including amino acid deprivation and mitochondrial stress. Such stress-induced eIF2α phosphorylation attenuates general mRNA translation and, at the same time, stimulates the preferential translation of specific downstream factors to orchestrate an adaptive gene expression program. In recent years, there have been significant new advances in our understanding of ISR during metabolic stress adaptation. Here, I discuss those advances, reviewing among others the ISR activation mechanisms in response to amino acid deprivation and mitochondrial stress. In addition, I review how ISR regulates the amino acid metabolic pathways and how changes in the ISR impact the physiology and pathology of various disease models.

Our cells frequently face stress imposed by physiological and environmental conditions that could cause cellular dysfunction and death. In response, robust cellular stress response mechanisms have evolved to enhance cellular adaptation. One such mechanism is the integrated stress response (ISR), which refers to signaling pathways activated upon phosphorylation of the translation initiation factor, eIF2α (1).

eIF2α phosphorylation occurs through kinases that respond to distinct conditions of cellular stress (Fig. 1). Four well-established eIF2α kinases share a high degree of sequence homology. These include protein kinase RNA-dependent (PKR), which is activated by double-stranded RNAs (2), and heme regulated inhibitor kinase (HRI) activated upon heme deprivation (3). PKR and HRI genes are conserved in vertebrates only. PKR-like ER kinase (PERK, also known as PEK or Pancreatic eIF2α Kinase) is an eIF2α kinase that localizes to the endoplasmic reticulum (ER) membrane, activated in response to ER stress (4, 5). PERK is broadly conserved in metazoans, including *Caenorhabditis elegans, Drosophila,* and mammals. Perhaps the most phylogenetically conserved is general control nonderepressible 2 (GCN2), an eIF2α kinase most famously activated by amino acid deprivation, conserved from yeast to humans (6, 7). In addition, two eIF2α kinases that do not share sequence homology with the other four established eIF2α kinases have been reported (8, 9). Taken together, the term ISR highlights the fact that seemingly distinct stress-activated kinases initiate a shared signaling output.

**Figure 1:**
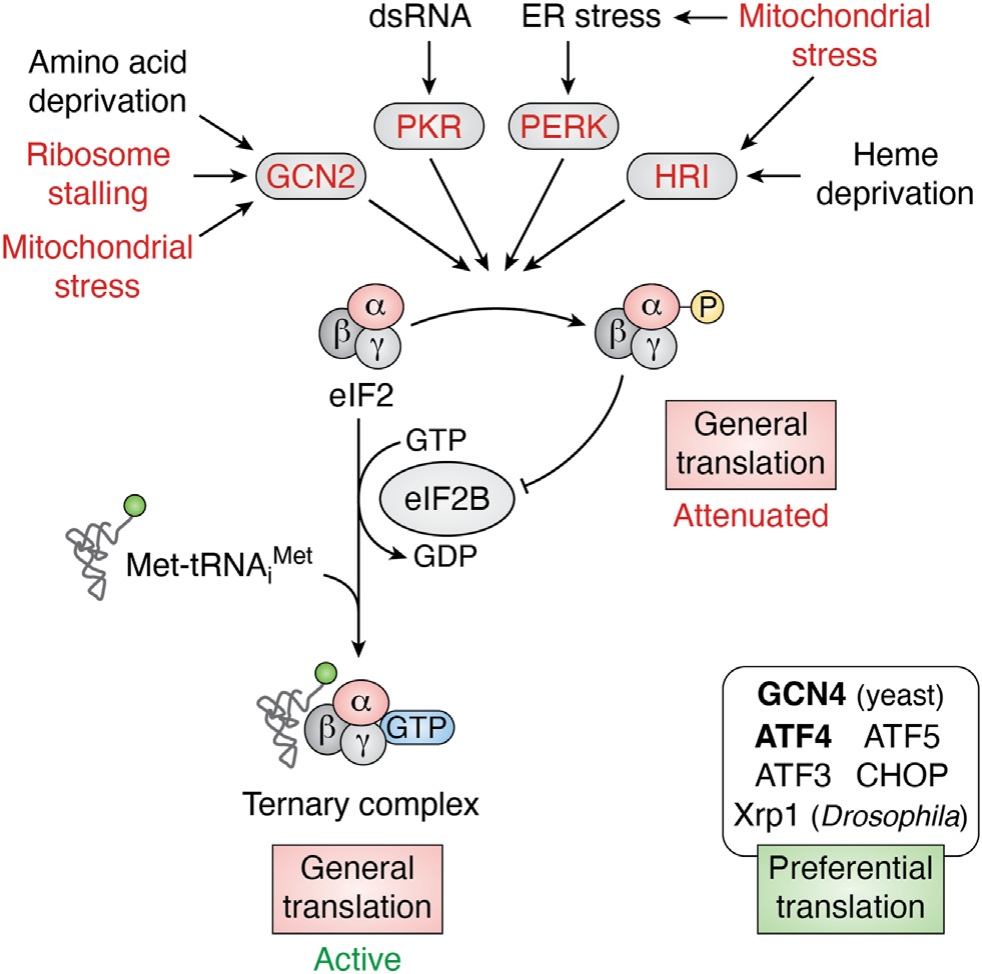
**The effect of eIF2α phosphorylation.** eIF2 consists of three subunits, α, β, and γ (α subunit is in *pink*), which form a ternary complex (TC) with GTP and Met-tRNA^i^_Met_ to assist translation initiation by the 40S ribosome subunit. Several stress-activated kinases can phosphorylate eIF2α, including GCN2, PKR, PERK, and HRI to suppress translation initiation. Recent studies found that ribosome stalling or mitochondrial stress can activate ISR through specific eIF2α kinases (*red*). Once phosphorylated, phospho-eIF2α acts as an allosteric inhibitor of the eIF2B complex. eIF2B’s normal role is to act as a GEF (guanine nucleotide exchange factor) for eIF2 to generate active TCs for mRNA translation. Therefore, the consequence of eIF2 phosphorylation is attenuation of general translation. At the same time, these conditions induce the preferential translation of a few downstream factors, including GCN4 (yeast) and ATF4 (metazoans) that induce stress-responsive gene transcription. GCN2, general control nonderepressible 2; ISR, integrated stress response; HRI, heme regulated inhibitor kinase; PERK, PKR-like ER kinase; PKR, protein kinase RNA-dependent.

Phosphorylation of eIF2α in response to the stress-activated kinases attenuates general mRNA translational attenuation. In addition, eIF2α phosphorylation stimulates the preferential translation of a few mRNAs, including some that encode transcription factors. Induction of those transcription factors during ISR activates a stress-responsive gene transcription program (1, 10, 11, 12) (Fig. 1).

ISR has drawn significant biomedical interest partly due to its association with many diseases. Reflecting ISR’s critical role in cellular homeostasis, loss of ISR has been associated with metabolic diseases (13, 14, 15, 16), pulmonary diseases (17), and diseases of the nervous system (18, 19). Conversely, excessive ISR signaling can contribute to the phenotypes of several disease models, including cancer (20, 21, 22), Alzheimer’s disease (23, 24), peripheral neuropathies (25, 26, 27, 28), and Down’s syndrome caused by chromosomal trisomy (29). In addition, blocking ISR can suppress cognitive deficits and memory decline in animal models (30, 31, 32). Studies of model organisms, including *C. elegans, Drosophila,* and mice, have contributed significantly to these studies, which will be reviewed in this article.

Notably, a number of studies published in the past 5 years have significantly changed our understanding of ISR’s relationship with metabolism. Specifically, these studies have advanced our view regarding how amino acid deprivation activates the ISR, how ISR responds to mitochondrial stress, and the significance of metabolic changes caused by ISR activation. In this review, I will first briefly introduce the basic pathway of ISR signaling and then highlight these recent advances regarding ISR signaling and metabolic adaptation.

## A brief summary of mRNA translation regulation during ISR

A defining feature of ISR is the phosphorylation of the ser51 residue within the α subunit of eIF2 that attenuates global mRNA translation. This initiates a sequence of mRNA regulatory mechanisms to induce the ISR, which has been reviewed extensively elsewhere (10, 33). Because this review will cover how metabolic stressors impact the mRNA translation machinery, I will briefly summarize the salient points here. In brief, eIF2 consists of α, β, and γ subunits and forms a ternary complex (TC) with GTP and Met-tRNA_i_^Met^ (Fig. 1). This TC associates with the 40S ribosomal subunit to form the 43S pre-initiation complex. With the assistance of other initiation factors, the pre-initiation complex scans the mRNA for an AUG start codon. Once the AUG start codon is recognized, the GTP of the TC is hydrolyzed to trigger the release of eIF2, now bound to GDP (10). To have eIF2 engage in a new round of translation initiation, eIF2 needs to re-acquire GTP through the action of its guanine nucleotide exchange factor, eIF2B (34) (Fig. 1). The phosphorylated form of eIF2’s α subunit inhibits general mRNA translation by acting as an inhibitor of eIF2B instead of being its substrate (10, 33, 35, 36, 37, 38, 39, 40, 41, 42) (Fig. 1). As a consequence, there is an attenuation in general mRNA translation.

eIF2α phosphorylation reduces general mRNA translation but also preferentially induces the translation of mRNAs encoding transcription factors, phosphatase subunits (PPP1R15A), membrane transporters (SLC35A4), cell cycle inhibitors (CDKN1A), and a BTB domain protein (IBTKα) (11, 43, 44, 45, 46). Many of these genes contain regulatory upstream ORFs (uORFs) that mediate their translational induction. Of note, uORFs are present in many mRNAs, but only a small fraction has such regulatory properties (reviewed in (47)).

An important factor that affects phospho-eIF2α levels is the phosphatase regulatory subunit, PPP1R15. In humans, there are two PPP1R15 genes; PPP1R15B (also known as CReP) expression is constant; PPP1R15A (also known as GADD34) is induced by ISR (48). Both, PPP1R15A and PPP1R15B help to dephosphorylate eIF2α and suppress ISR (49, 50). Many studies in various models inhibit PPP1R15 to activate ISR signaling (50, 51, 52, 53).

Another conserved ISR target across phyla is the translational inhibitor 4E-BP1 and its *Drosophila* homolog *Thor* (54, 55). 4E-BPs bind and inhibit eIF4E, a cap-binding initiation factor that helps load ribosomes to most cellular mRNAs (33, 56). Intriguingly, 4E-BP helps to enhance stress resistance in various model organisms (57, 58, 59). While 4E-BP dampens the translation of many cellular transcripts, several stress response genes contain internal ribosome entry sites to bypass translational inhibition caused by 4E-BP (55, 60, 61, 62, 63, 64), which may contribute to stress adaptation.

Recent studies have identified a number of lesser-known initiation factors involved in ISR regulation. For example, eIF2D and DENR/MCT-1 are proteins with eIF2-like activities *in vitro*, which are able to deliver aminoacyl-tRNAs to the P-site of the ribosome (65, 66). Recent studies found that eIF2D and DENR/MCT-1 are required for ATF4 induction in *Drosophila* and humans (67, 68). In addition, a noncanonical cap-binding protein, eIF3d, is required for ISR signaling under chronic stress (69). eIF3d specifically contributes to the translation of GCN2 in cells and affects ATF4 translation indirectly through an m6A demethylase (70). These studies highlight some of the unconventional translation regulatory mechanisms involved in ISR signaling.

### Transcription factors preferentially translated upon eIF2α phosphorylation

Transcription factors are among those that undergo preferential translation in response to eIF2α phosphorylation. Yeast GCN4 was the first such transcription factor to be identified, whose mRNA has a regulatory 5’ leader (also referred to as 5’ UTR). This 5’ leader contains regulatory uORFs that precede the main GCN4 ORF. The detailed underlying mechanism of GCN4 translation in response to eIF2α phosphorylation has been extensively reviewed elsewhere (10, 47, 71, 72).

There are now several metazoan transcription factors known to have regulatory 5’ leaders that allow their induction during ISR. Perhaps the most extensively characterized is the bZIP transcription factor, ATF4 (73, 74). In addition, a small number of other bZIP transcription factors, including ATF3, ATF5, and CHOP (also known as DDIT3), contain 5’ leaders that allow their preferential translation in response to eIF2α phosphorylation (73, 75, 76). ATF4 and CHOP together regulate the expression of many common target genes, including PPP1R15A (77). The ATF4–CHOP–PPP1R15A axis of ISR helps cells restore the overall protein synthesis capacity, but this pathway can promote cell death under conditions of chronic ISR activation (49, 77). Although not part of the bZIP family, QRICH1 was recently identified as another DNA-binding protein induced during ISR (78).

Invertebrate model organisms also have ISR-mediating transcription factors with similar regulatory 5′ leaders. These include the *Drosophila* ATF4 homolog *cryptocephal* (crc), which plays critical roles in stress adaptation (51, 55, 79). The status of *crc* as the lone ISR mediator has changed recently with the recognition of another bZIP transcription factor, Xrp1, that is induced in response to eIF2α phosphorylation and mediates the stress response associated with ribosome subunit heterozygosity (53, 80). The *C. elegans, atf-4* (also known as *atf-5*) mRNA similarly undergoes preferential translation in response to ER stress or when they are challenged with protein synthesis inhibitors or mTORC1 RNAi. Highlighting the importance of enhanced stress adaptation, *C. elegans atf-4* induction can help extend the lifespan of this organism (81).

### ISR target genes that regulate amino acid metabolism

ISR target genes have been extensively profiled in various cell types and organisms. While there is some degree of cell type specificity, there are also shared target genes generally conserved across cell types and phyla. While these include various aminoacyl tRNA synthetases, quality control genes in the ER, mitochondria, and the anti-oxidant response (51, 74, 77, 82), here I will highlight specifically those involved in amino acid metabolism.

**Figure 2:**
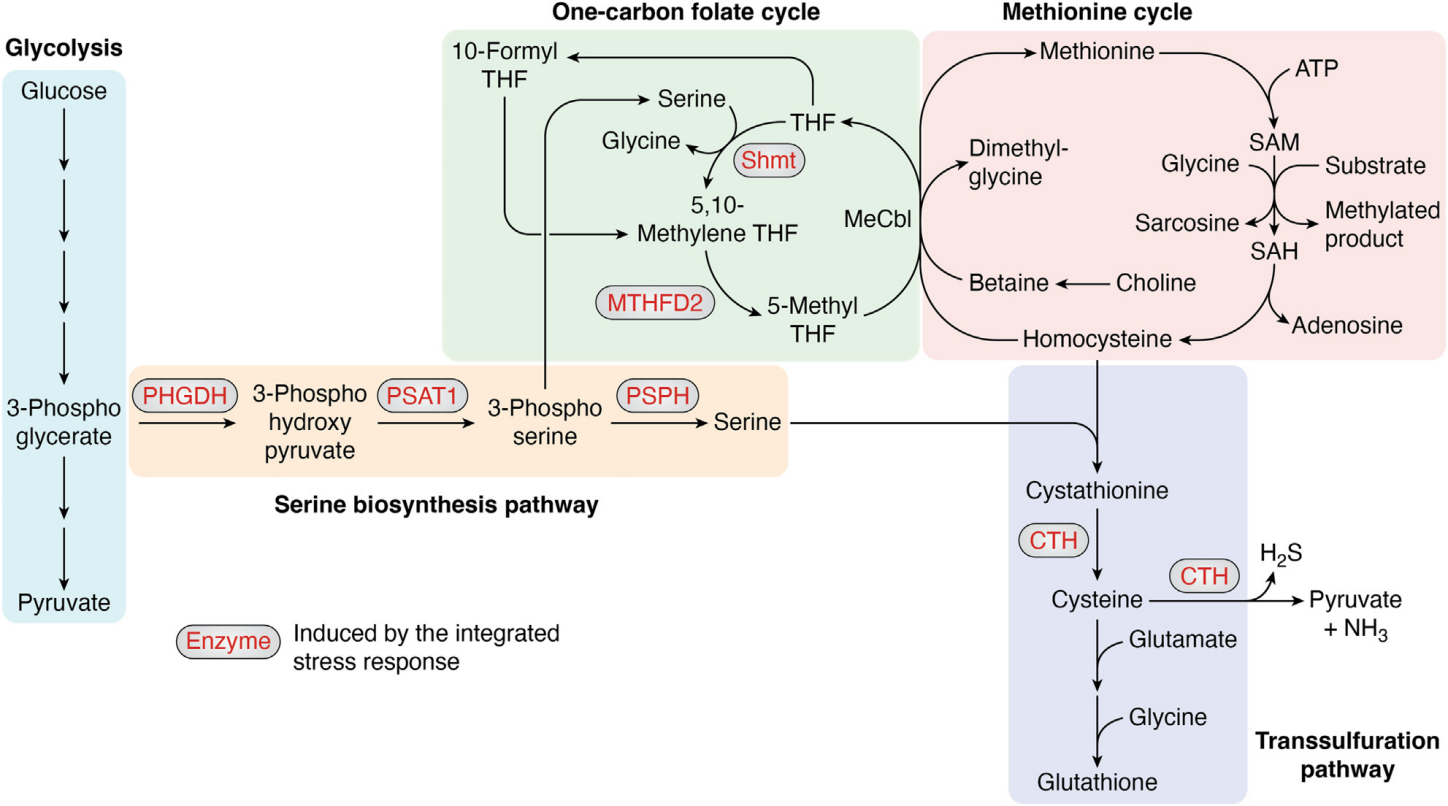
**ISR induces enzymes that mediate amino acid biosynthesis.** A schematic diagram of metabolic pathways stimulated by ISR. Specifically highlighted are glycolysis (*blue* background), serine biosynthesis (*yellow* background), one-carbon folate cycle (*green* background), methionine cycle (*pink* background), and the transsulfuration pathways (*gray* background). The enzymes induced by ISR are shown in *red* characters in boxes. Specifically, the ISR-mediating transcription factors ATF3 and ATF4 induce PHGDH, PSAT1, PSPH, MTHFD2, and SHMT. ATF4 also induces CTH and enzyme that catalyzes cysteine biosynthesis. The net effect of ISR induction is to divert a glycolysis intermediate (3-phospho glycerate) to produce more amino acids and nucleotides. Cysteine is also used to produce glutathione, a major antioxidant molecule in cells. ISR, integrated stress response.

An important sub-class of ISR targets include those genes that promote amino acid biosynthesis and the one-carbon folate cycle (83, 84, 85) (Fig. 2). ATF4 and ATF3 induce PHGDH, PSAT1, and PSPH, which encode enzymes involved in converting a glycolysis intermediate, phosphoglycerate, to serine (77, 83, 86). The net effect of inducing these enzymes is to increase amino acid levels at the expense of energy production from glucose metabolism. In a subsequent step, the enzyme Shmt converts serine into glycine and transfers the methyl group from serine to the one-carbon folate cycle. Enzymes in the folate cycle, including Shmt and MTHFD2, are also induced by ATF4, ATF3, and CHOP (87, 88, 89, 90). The products of the folate cycle serve as precursors for nucleotide biosynthesis, required for cell proliferation and DNA replication. Consistent with ISR’s role in promoting the biosynthesis of amino acids and nucleotides, the ATF4-folate cycle contributes to tumor growth (91, 92, 93). Other transcription factors of the ISR, including ATF3 and CHOP, also modulate this metabolic response in models of leukemia and mitochondrial cardiomyopathy (90, 94).

The ATF4-stimulated metabolic pathways also contribute to the maintenance of cellular redox potential. This is partly because ATF4 promotes the biosynthesis of cysteine, which plays critical roles against cellular oxidants. Cysteine exerts its anti-oxidant effect partly as a component of glutathione (consisting of glutamate, cysteine, and glycine). In this context, the cysteine residue’s thiol group (-SH) serves as an important electron donor to maintain a reducing cellular environment. ATF4 induces the transcription of multiple enzymes involved in cysteine biosynthesis. For one, serine is a precursor for cysteine synthesis. In addition, ATF4 induces cystathionine gamma-lyase (known as CTH, CGL, or CSE) (84, 95, 96), which converts homocysteine to cysteine (Fig. 2). Homocysteine is a metabolite of the methionine cycle, which uses 5-methyl tetrahydrofolate from the one-carbon folate cycle. Therefore, the combined effect of ATF4 stimulation is to promote the production of amino acids that make up glutathione.

A recent study has identified an additional link between ATF4 and an antioxidant mechanism related to this pathway. Specifically, stimulating ATF4 in *C. elegans* produces high levels of hydrogen sulfide (H_2_S) through the enzyme CTH that mediates cysteine metabolism (Fig. 2). This ATF4–H_2_S axis helps to prolong *C. elegans* lifespan by reducing oxidized thiol groups (specifically sulfenic acids, -SOH) in a process called persulfidation (81). The protective effect of ATF4 against oxidation is not limited to *C. elegans.* ATF4 KO mouse embryonics fibroblasts (MEF) cells are vulnerable to oxidative stress (74). Among the *Drosophila* models of Parkinson’s Disease are *Parkin* or *Pink* mutants. These mutants have dysfunctional mitochondria and oxidative stress, and the ATF4-folate metabolism helps to suppress various phenotypes associated with the mutants, including the loss of dopaminergic neurons (88). These studies support an evolutionarily conserved role of ATF4 in promoting amino acid biosynthesis and the maintenance of cellular redox potential.

### The mechanisms of GCN2 activation during amino acid deprivation

**Figure 3:**
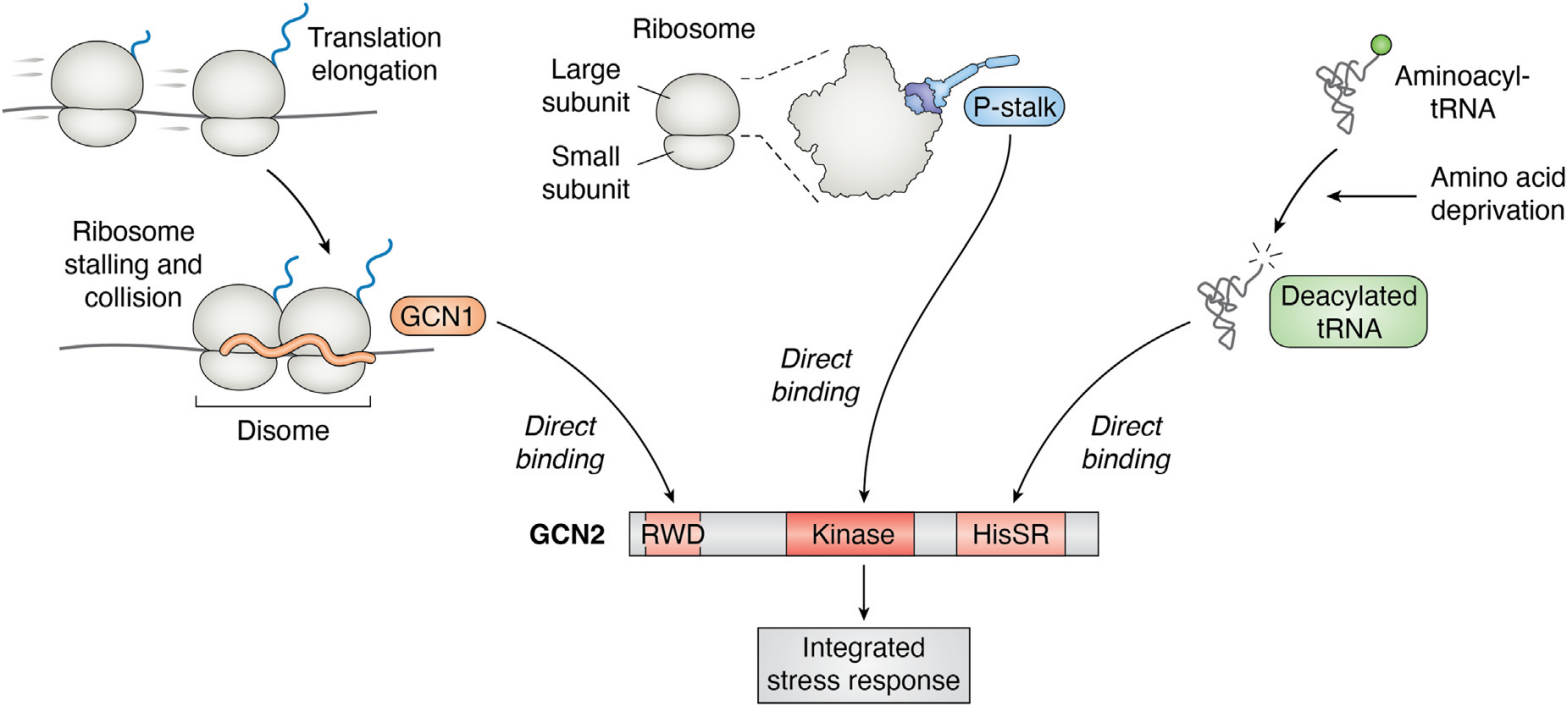
**Regulation of GCN1 and GCN2.** Shown are three different mechanisms of GCN2 activation. (*Right*) Deacylated tRNAs that increase upon amino acid starvation bind to the HisRS domain of GCN2 to stimulate the kinase activity. (*Center*) Ribosome subunits that make up the P-stalk can bind and activate the GCN2 kinase. (*Left*) Ribosome stalling during mRNA translation generates collided “disomes”, which binds to GCN1 to activate GCN2. GCN2, general control nonderepressible 2.

ATF4 may induce amino acid metabolism genes as a homeostatic mechanism against amino acid deprivation. GCN2 responds to amino acid deprivation, and the mechanism of its activation has been an active area of research. Early studies focused on the role of deacylated (those uncharged with cognate amino acids) tRNAs, whose levels increase in cells when amino acids are deprived (97, 98, 99). GCN2 has a domain homologous to the histidyl tRNA synthetase (HisRS-like), which can bind to deacylated tRNAs directly to stimulate GCN2’s kinase activity (6, 97, 100, 101) (Fig. 3).

Recent studies have uncovered alternative mechanisms for GCN2 activation. One study found that GCN2 is more potently activated *in vitro* by ribosome subunits that comprise the P-stalk. Direct interaction with the P-stalk subunits prompts GCN2’s HisRS and kinase domains to undergo conformational changes for kinase activation (102) (Fig. 3). The GCN2 HisRS-like domain, implicated in tRNA binding, contributes to the GCN2–ribosome interaction. The P-stalk region is well known for its ability to interact with translation elongation factors, and therefore, the findings imply that abnormal translation elongation triggers GCN2 activation. Independent studies confirmed that deletion of P-stalk subunits attenuated GCN2 activation in yeast and mammalian cultured cells (103, 104).

The two models involving uncharged tRNA and P-stalk suggest regulatory roles of HisRS. The two models also indicate the importance of GCN2 interaction with the ribosome. In the P-stalk model, the direct interaction without the aid of other proteins is sufficient to activate GCN2 *in vitro*. In the model involving uncharged tRNAs, GCN2 interacts with ribosomes together with co-factors that include GCN1 and GCN20 (105, 106).

A distinct GCN2 activation model proposes that problems in translational elongation cause ribosomes to stall and collide on mRNAs, and the resulting emergence of two juxtaposed ribosomes (disomes) triggers the activation of GCN2 (Fig. 3). This idea first emerged from the analysis of a mutagenized mouse strain called *nmf205* that lacks a nervous system–specific tRNA together with GTPBP2, whose normal role is to help dissociate collided ribosomes on mRNAs (referred to as ribosome rescue). As a result, the cerebellar cells of these mice show an increased degree of ribosome stalling on mRNAs (107), which activates GCN2-mediated ISR signaling (108, 109). One may wonder why multiple mechanisms of GCN2 activation have evolved. While experimental conditions can cause uncharged tRNA buildup in cultured cells, dietary restriction of amino acids in mice does not (110). The difficulty of building up uncharged tRNA may be due to interorgan metabolism and amino acid recycling. The alternative mechanisms of GCN2 activation may allow more efficient ISR activation in whole animal tissues.

Both GCN2 and GTPBP2 play protective roles in the *nmf205* model of mouse neurodegeneration with excessive ribosome stalling (108). Interestingly, previous genomic studies have found that GTPBP2 is a target of the ISR transcription factors ATF4 and CHOP (77). Taken together, these results suggest that the GCN2–ATF4–GTPBP2 axis is induced by widespread ribosome stalling, and this pathway forms a negative feedback loop to help rescue stalled ribosomes from mRNAs. Supporting this idea, blocking ISR signaling enhances the degree of collided ribosomes on mRNAs (111).

### A complex relationship between GCN2 and the amino acid deprivation response

With the emergence of different GCN2 activation mechanisms, two recent studies reported that some mediate GCN2 activation independent of amino acid deprivation. Specifically, the studies created conditions in yeast where deacylated tRNA levels remained unchanged while increasing the degree of ribosome stalling. These conditions activated GCN2 in yeast and is referred to as “amino acid starvation–independent ribosome stalling” (104, 112).

The studies on GCN2 activation mechanisms have prompted an in-depth examination of two additional factors, GCN1 and GCN20, which are required for GCN2 activation (106, 113, 114). For example, recent studies indicate that GCN1 is required for both the amino acid starvation response and the amino acid starvation–independent ribosome stalling response (104, 112). On the other hand, ribosome P-stalk subunits reportedly have a more specific role in mediating GCN2 activation after excessive ribosome stalling, after osmotic stress, or glucose deprivation. Interestingly, P-stalk mutants did not mediate GCN2 upon deprivation of specific amino acids, implicating its role in an amino acid starving–independent GCN2 activation (104).

Even when we restrict our focus to the amino acid deprivation response, the literature reports GCN2-independent mechanisms of ISR activation. An example of this case is found in mice deprived of dietary sulfur amino acids. The livers of these mice showed significant ATF4 induction. Surprisingly, such ATF4 pathway induction was independent of GCN2 (110, 115, 116). An independent study in *Drosophila* larvae found that ISR is most sensitively activated in response to tyrosine deficiency in the diet. Surprisingly, ISR induction by tyrosine deficiency did not require GCN2 (117).

Together, these studies suggest that GCN2 activation is not always due to the amino acid deprivation response. There are exceptions to the old rule. GCN2 now has established roles unrelated to the amino acid deprivation response. Conversely, certain conditions of amino acid deprivation do not activate GCN2.

### GCN1 is a signaling hub

The precise relationship between GCN1 and collided ribosomes had remained unclear until the recent elucidation of a cryo-EM structure of the yeast GCN1–GCN20 ribosome complex. In that structure, GCN1 was found to form an elongated structure that spans two 80S ribosomes juxtaposed to each other, suggesting that GCN1 acts as a sensor for collided ribosomes (118) (Fig. 3).

There is evidence that collided ribosomes and GCN1 serve as a platform for the intersection of several stress-response mechanisms, including the ribosome-associated quality control (RQC) pathway. The RQC pathway is initiated by ribosome stalling on mRNA to degrade newly synthesized peptides through the proteasome (119). An example of RQC action occurs when ribosomes fail to terminate translation on stop codons and continue mRNA translation into the polyA sequence before being stalled. The resulting collision of two ribosomes creates an interface that is recognized by Hel2 (yeast) or ZNF598 (mammals), which are ubiquitin ligases that ubiquitinate the 40S ribosomal subunit to initiate ribosome rescue (120, 121, 122). This RQC mechanism antagonizes the ISR, as the loss of Hel2 increases collided ribosomes and activates the GCN2 pathway (112, 123).

Studies indicate that GCN1 physically interacts with several other proteins involved in stress response. For one, GCN1 recruits the CCR4/NOT nuclease complex to degrade mRNAs with stalled ribosomes (124) (Fig. 2*B*). GCN1 also recruits the E3 ubiquitin ligase, RNF14, to help degrade the elongation factor eEF1A on stalled ribosomes (125). In addition, GCN1 on the collided ribosomes interacts with ZAKα, an upstream kinase for p38 and JNK. The loss of ZAKα somehow reduces eIF2α phosphorylation and suppresses the p38–JNK pathway (111). These results indicate that GCN1 serves as a critical general sensor of collided ribosomes.

A recent mouse knockout study has provided added insights regarding the relationship between GCN1 and GCN2. As expected, GCN1 KO mouse fibroblasts could not induce ISR in response to amino acid deprivation. In addition, GCN1 KO mice exhibited additional developmental defects not found in GCN2 null mice (126). These results are consistent with the recent mechanistic studies supporting a broader role of GCN1 in mediating several distinct stress response pathways.

### eIF2α kinases that respond to mitochondrial stress

Mitochondria is an essential subcellular organelle for energy production and cellular metabolism. Many ATF4-inducible genes, including Shmt2 and MTHFD2, regulate metabolism in the mitochondria. At the same time, dysfunctional mitochondria can reduce the biosynthesis of certain metabolites, including specific amino acids, and produce reactive oxygen species. In response, several established signaling pathways are activated to regulate nuclear gene transcription. These pathways are sometimes referred to as “mitochondrial retrograde signaling” or the mitochondrial unfolded protein response (mtUPR). Early studies on the mtUPR pathway in *C. elegans* had examined the transcription factor ATFS-1, which is regulated through a unique mechanism (127). The mammalian gene with the closest sequence homology to ATFS-1 is ATF4. In mammals, ISR plays an equivalent role as a central mediator of mtUPR (85, 128, 129, 130, 131, 132, 133). More recent studies have implicated several different eIF2α kinases in the mitochondrial stress response, depending on cell types and the specific nature of the mitochondrial stress.

Some studies have reported that HRI serves as the eIF2α kinase mediating ISR activation after the disruption of mitochondrial function (132, 134, 135). HRI was originally characterized as a kinase activated upon Heme deprivation (136). HRI resides in the cytoplasm, and therefore, its activation would require a signaling factor from the mitochondria to mediate the mitochondrial stress response. Adding the well-established HRI-regulatory molecule, Heme, did not affect ISR activation by mitochondrial stress. Instead, evidence indicated that mitochondrial dysfunction activates the metalloprotease OMA1, which cleaves DELE1. The cleaved DELE1 is released to the cytoplasm, undergoes oligomerization, and directly binds HRI to stimulate eIF2α phosphorylation and ISR activation (132, 134, 137). This pathway is further fine-tuned by UBR4, a large cytoplasmic ubiquitin ligase that promotes the degradation of cleaved DELE1 and HRI. Cleaved DELE1 and HRI compete with other mitochondrial proteins that accumulate in the cytoplasm. Through this competition mechanism, other mitochondrial proteins in the cytoplasm may titrate away UBR4 and stabilize DELE1 and HRI, which may cause a sustained ISR signaling until the stress is resolved (138).

Other studies support the role of PERK in mediating the mitochondrial stress response. One form of mitochondrial stress could be caused by mutations in Pink1 and Parkin, which underlie rare forms of early-onset Parkinson’s Disease (139, 140). At the molecular level, Pink1 and Parkin proteins help degrade damaged mitochondria through mitophagy, and therefore, loss of these genes causes the buildup of dysfunctional mitochondria in cells (141, 142, 143, 144). Studies in *Drosophila* indicate that the loss of Pink1 or Parkin activates ISR through PERK, and the knockdown of PERK in these flies increases the survival of dopaminergic neurons (88, 145). ER-localized PERK may respond to mitochondrial stress through the mitochondria-ER contact sites because weakening that contact site through Mitofusin RNAi reduces ISR activation in these flies (88). An independent study examining a *Drosophila* tumor model found additional support for PERK in the mitochondrial stress response. In this model, conditions that inhibited the mitochondrial electron transport chain promoted Notch-induced tumor growth, and PERK was required for such tissue overgrowth (146).

PERK’s role in the mitochondrial stress response may not be limited to invertebrates. In mammals, active PERK helps to degrade components of the mitochondrial protein import machinery, thereby restricting the buildup of damaged or non-native proteins into the mitochondria (147). Furthermore, PERK activation protects against pathologic mitochondrial fragmentation, in part, by remodeling the mitochondrial membrane phosphatidic acids (148, 149).

GCN2 has also been shown to mediate ISR induction in response to inhibition of the mitochondrial electron transport chain. Such conditions reduce NADH, which lowers asparagine levels to activate GCN2 (133). Interestingly, the authors have found that this relationship between the mitochondrial inhibitor and ISR is cell type–specific. This study demonstrates that there are many different types of mitochondrial stress, and cells use different eIF2α kinases dependent on cell types and the nature of the mitochondrial dysfunction.

### eIF2α kinase-independent ISR activation

ISR has been defined as a pathway activated in response to eIF2α phosphorylation. But there is now a growing number of examples where this pathway becomes active without the involvement of eIF2α kinase. First, I will highlight two classes of pathological conditions.

One class is a set of mutations in the eIF2B complex that underlie Vanishing White Matter (WWM) disease. Even without eIF2α phosphorylation, the mutations impair eIF2B function to activate ISR, which drives the loss of the CNS white matter (150). Recently, a natural compound stabilizing a mutant eIF2B complex has been identified (151). The existence of a natural metabolite that affects eIF2B and ISR signaling further raises the possibility that cells actively regulate ISR through eIF2B and bypass the requirement of eIF2α kinases.

Another example is the X-linked intellectual disability syndrome, MEHMO, caused by missense mutations in the eIF2S3 gene encoding the eIF2γ subunit (152). The mutation impairs Met-tRNA_i_^Met^ binding to eIF2 (153), constitutively activates ISR, and interferes with neuronal differentiation in patient-derived iPSC cells. Pharmacological inhibition of ISR activation suppresses these phenotypes (154).

Other studies report that mTORC1 can regulate ISR independent of eIF2α kinases. In cultured MEF cells, activation of mTORC1 induces the ATF4 pathway even in cells with mutant eIF2α that cannot be phosphorylated (87, 155). An independent study reported that mTORC1 regulates both ATF4 mRNA stability and mRNA translation. mTORC1 regulates mRNA translation in part by inhibiting 4E-BP, which results in more active eIF4E. The study concluded that enhanced eIF4E function contributed to increased ATF4 mRNA translation (156). This mTORC1–ATF4 axis allows growth factors to enhance ATF4 target gene expression involved in purine biosynthesis, amino acid uptake, and amino acid biosynthesis.

Of note, there are now numerous studies reporting the varying relationships between mTORC1 and ATF4. While the above-mentioned studies in cultured MEF and HEK293 cells support ATF4 activation by mTORC1, studies in yeast and *C. elegans* have reported an opposite relationship where mTORC1 exerts an inhibitory effect on ATF4 and GCN4 (81, 157).

There are additional regulatory inputs into the ISR unrelated to eIF2α regulation. A study based on yeast has shed light on the regulatory mechanism impinging on GCN4 stability. In this organism, high levels of methionine in the media increase GCN4 levels. This does not occur through eIF2α kinase stimulation but instead by a signaling cascade that involves SAM that increases GCN4 stability (158). If there is a basal level of eIF2α kinase that produces a small amount of unstable GCN4, regulatory inputs that stabilize this transcription factor could effectively stimulate ISR signaling. These studies highlight that ISR can be regulated through diverse mechanisms and do not necessarily require changes in eIF2α kinase activity.

The increasing literature on the eIF2α kinase–independent ISR regulation prompts us to reflect on the older claims about the central role of eIF2α kinases. One study had knocked out four conserved eIF2α kinases in cultured cells and found that these cells are unable to induce ISR in response to 12 different chemical stressors (159). It is possible that the eIF2α kinase–independent mechanisms are relatively minor as compared to ISR activation by eIF2α kinases. Alternatively, it is also possible that the newly identified eIF2α kinase–independent mechanisms were not represented among the specific conditions examined in the earlier studies.

## Concluding remarks

The critical importance of ISR in cellular quality control, translation regulation, and disease implications has been well established. At the same time, recent studies have uncovered new relationships between ISR and its response to amino acid deprivation and mitochondrial stress. The latest findings support the idea that ISR integrates an even broader set of stress conditions through previously unknown mechanisms. Since a significant part of amino acid metabolism relies on the mitochondria, signaling outputs of the ISR may impact the adaptation to both conditions.

## Conflict of interest

The author declares no conflict of interest within the content of this article.
